# Supplementary material for: Effect of Borrelia burgdorferi Outer Membrane Vesicles on Host Oxidative Stress Response
Source: Antibiotics (Basel). 2020 May 25;9(5):275. doi: 10.3390/antibiotics9050275 (PMC7277464; doi:10.3390/antibiotics9050275)
Supplement: Supplementary file 1 [file antibiotics-09-00275-s001.pdf]

Supplementary material

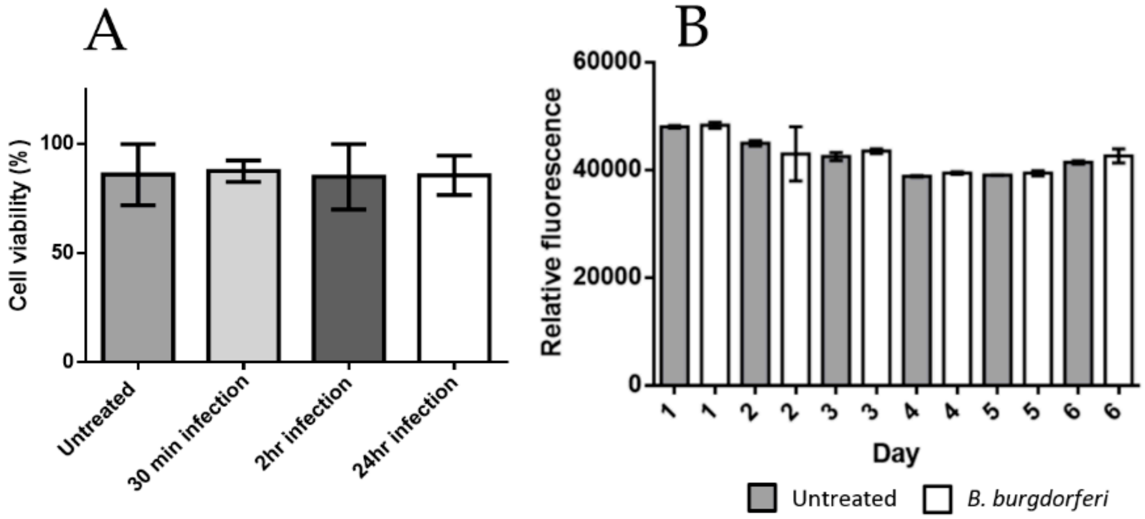

Figure 1. BE2C cell viability after co-culture with *B. burgdorferi*. BE2C cells were co-cultured with *B. burgdorferi* for 30 min, 2 h, and 24 h timepoints, after which viability was assessed using trypan blue (**A**). Additionally, BE2C cell viability after *B. burgdorferi* exposure from 1 to 6 days was assessed using Alamar blue (**B**). Error bars represent SEM. Trypan blue staining was performed by diluting equal parts BE2C cells Trypan blue. Live and dead cell counting was performed using a hemocytometer. An Alamar blue assay (Thermo Fisher Sci, Cat. no. DAL1100, Waltham, MA) was performed by seeding 15,000 BE2C cells per well in a 24 well plate. Infections were carried out in triplicate from 1-6 days. Alamar blue was added according to the manufacturer's recommendations at the appropriate timepoint. Readings were made 24 h after addition of Alamar blue using a Bio-tek FLX800 plate reader. No significant differences in viability were observed at any infection time point.

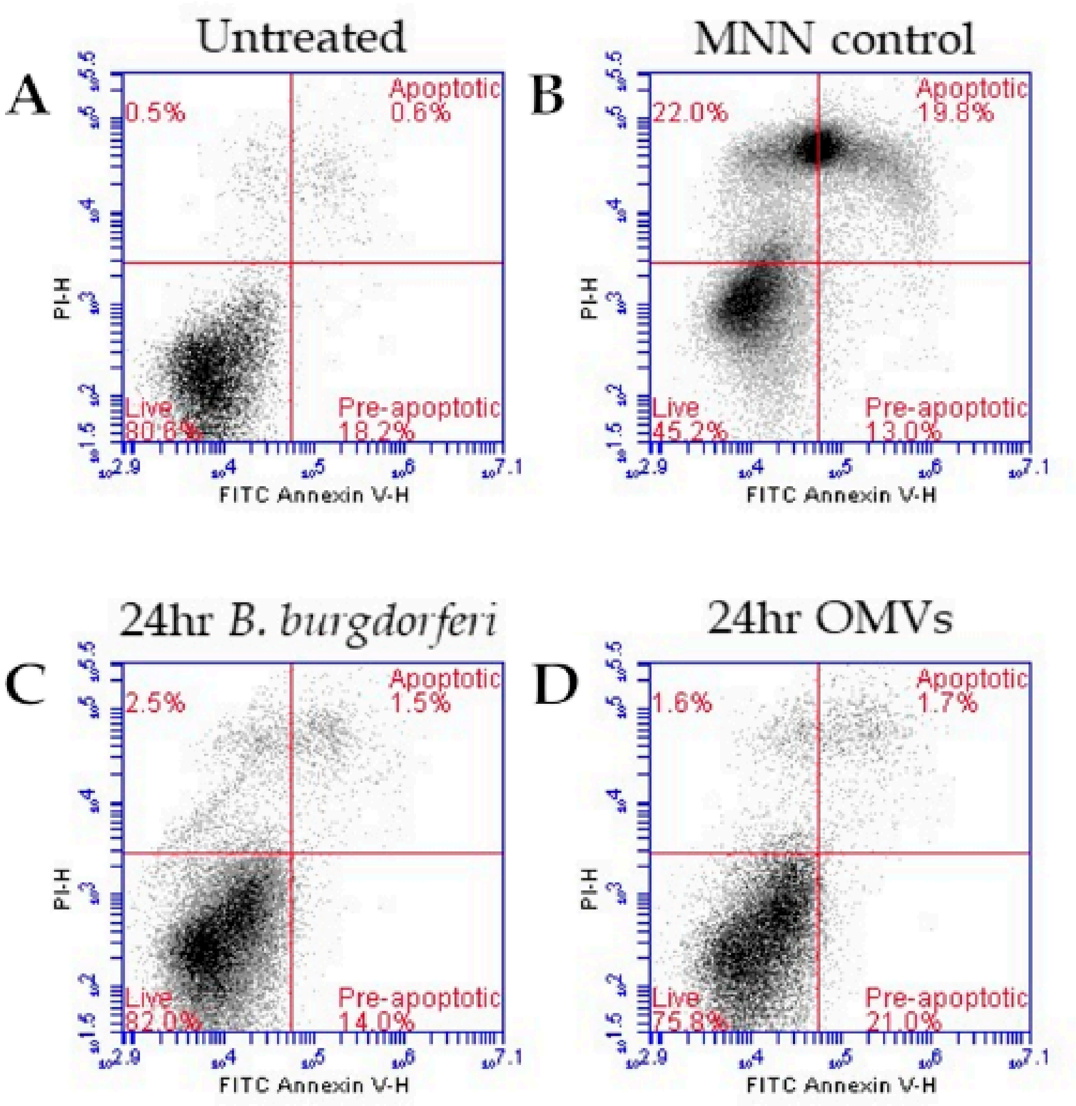

Figure 2. Apoptosis in BE2C cells after 24 hours *B. burgdorferi* or OMV co-culture. After exposure to *B. burgdorferi* or OMVs, BE2C cells were stained with PI and FITC-annexin V and assessed on a BD Accuri C6 flow cytometer. Untreated cells were stained with FITC-annexin V (**A**). Menadione (MNN) was used as a positive control to induce apoptosis (**B**). Cells co-cultured with *B. burgdorferi* (**C**) or OMVs (**D**) for 24 hours showed elevated levels of annexin/PI positive cells compared to the untreated control cells. Mitochondrial SOD2 activity is necessary for a cell's ability to keep oxidative stress in check [1]. Therefore, interruption of normal SOD2 activity leaves cells susceptible to DNA, protein, and lipid membrane damage followed by apoptosis [2]. The reduction in SOD2 observed in this study pointed to potential apoptotic pathway activation in BE2C cells. Gates for regions of no FITC/PI staining (live cells), and positive FITC/PI (apoptotic cells) were established using untreated and MNN control groups, respectively. BE2C cells exposed to *B. burgdorferi* for 24 hours showed 0.9% more apoptotic cells than untreated cells (Figure S2C), while exposure to OMVs proportional to the amount *B. burgdorferi* spirochetes had 1% more apoptotic cells (Figure S2D).

Reference

1. Kokoszka, J.E.; Coskun, P.; Esposito, L.A.; Wallace, D.C. Increased mitochondrial oxidative stress in the Sod2 (+/−) mouse results in the age-related decline of mitochondrial function culminating in increased apoptosis. *Proc. Natl. Acad. Sci. USA* **2001**, *98*, 2278–2283.
2. Sies, H.; Berndt, C.; Jones, D.P. Oxidative Stress. *Annu. Rev. Biochem.* **2017**, *86*, 715–748.
